# Special Notice

**Published:** 1892-11

**Authors:** 


### SPECIAL NOTICE.

Beginning with our next number—by the by, it will be an extra, or Christmas edition—there will be established a Department of Anatomy and Pathology, which will be under the management of Prof. Wm. Keiller, M. D., Professor of Anatomy in the Texas Medical College. Professor Keiller has kindly consented to contribute original articles to that department, illustrated with drawings by himself; and to report abstracts from the European journals.

A Department of Dermatology, which will be in charge of Dr. Isadore Dyer, of New Orleans, Lecturer and Clinical Instructor in Dermatology in Tulane University and Dermatologist to the Charity Hospital.

Also a Department of Clinical Reports from Charity Hospital, in charge of Dr. Robert Morris, Texas Resident Student at the Charity Hospital.

Dr. E. J. Beall, of Fort Worth, will spend the winter in Philadelphia, studying, especially, gynecology and the methods of the great operators in the several hospitals, and will contribute regularly to the pages of the JOURNAL.

Dr. R. H. L. Bibb is our Mexican Associate Editor and collaborator.

No pains will be spared to make the JOURNAL not only interesting and valuable to all who would keep up with medicine, but indispensable to the Texas profession. Meantime, an invitation is extended to Texas physicians to contribute their experiences to the JOURNAL for the benefit of their brethren.

